# Association Between Type 2 Diabetes and Changes in Myocardial Structure, Contractile Function, Energetics, and Blood Flow Before and After Aortic Valve Replacement in Patients With Severe Aortic Stenosis

**DOI:** 10.1161/CIRCULATIONAHA.122.063444

**Published:** 2023-09-25

**Authors:** Nicholas Jex, John P. Greenwood, Richard M. Cubbon, Oliver J. Rider, Amrit Chowdhary, Sharmaine Thirunavukarasu, Sindhoora Kotha, Marilena Giannoudi, Anna McGrane, Amanda Maccannell, Marcella Conning-Rowland, Sam Straw, Henry Procter, Sotiris Papaspyros, Betsy Evans, Kalyana Javangula, Antonella Ferrara, Walid Elmahdy, Pankaj Kaul, Hui Xue, Peter Swoboda, Peter Kellman, Ladislav Valkovič, Lee Roberts, David Beech, Mark T. Kearney, Sven Plein, Marc R. Dweck, Eylem Levelt

**Affiliations:** 1 University of Leeds, Multidisciplinary Cardiovascular Research Centre, and Biomedical Imaging Science Department, Leeds Institute of Cardiovascular and Metabolic Medicine, Leeds, UK (N.J., J.P.G., R.M.C., A.C., S.T., S.K., M.G., A. McGrane, A. Maccannell, M.C.-R., S.S., H.P., P.S., L.R., D.B., M.T.K., S.P., E.L.).; 2 Leeds Teaching Hospitals NHS Trust, Department of Cardiology, Leeds, UK (N.J., J.P.G., R.M.C., A.C., S.T., S.K., M.G., S.S., H.P., S.P., B.E., K.J., A.F., W.E., P. Kaul, P.S., M.T.K., E.L.).; 3 Oxford Centre for Clinical Magnetic Resonance Research (OCMR), RDM Cardiovascular Medicine, University of Oxford, UK (O.J.R., L.V.).; 4 National Heart, Lung, and Blood Institute, National Institutes of Health, Department of Health and Human Services, Bethesda, MD (H.X., P. Kellman).; 5 Department of Imaging Methods, Institute of Measurement Science, Slovak Academy of Sciences, Bratislava, Slovakia (L.V.).; 6 University of Edinburgh/BHF Centre for Cardiovascular Science, Edinburgh, UK (M.R.D.).

**Keywords:** aortic valve stenosis, diabetes mellitus, type 2, magnetic resonance imaging, myocardial blood flow

## Abstract

**BACKGROUND:**

Type 2 diabetes (T2D) is associated with an increased risk of left ventricular dysfunction after aortic valve replacement (AVR) in patients with severe aortic stenosis (AS). Persistent impairments in myocardial energetics and myocardial blood flow (MBF) may underpin this observation. Using phosphorus magnetic resonance spectroscopy and cardiovascular magnetic resonance, this study tested the hypothesis that patients with severe AS and T2D (AS-T2D) would have impaired myocardial energetics as reflected by the phosphocreatine to ATP ratio (PCr/ATP) and vasodilator stress MBF compared with patients with AS without T2D (AS-noT2D), and that these differences would persist after AVR.

**METHODS:**

Ninety-five patients with severe AS without coronary artery disease awaiting AVR (30 AS-T2D and 65 AS-noT2D) were recruited (mean, 71 years of age [95% CI, 69, 73]; 34 [37%] women). Thirty demographically matched healthy volunteers (HVs) and 30 patients with T2D without AS (T2D controls) were controls. One month before and 6 months after AVR, cardiac PCr/ATP, adenosine stress MBF, global longitudinal strain, NT-proBNP (N-terminal pro-B-type natriuretic peptide), and 6-minute walk distance were assessed in patients with AS. T2D controls underwent identical assessments at baseline and 6-month follow-up. HVs were assessed once and did not undergo 6-minute walk testing.

**RESULTS:**

Compared with HVs, patients with AS (AS-T2D and AS-noT2D combined) showed impairment in PCr/ATP (mean [95% CI]; HVs, 2.15 [1.89, 2.34]; AS, 1.66 [1.56, 1.75]; *P*<0.0001) and vasodilator stress MBF (HVs, 2.11 mL min g [1.89, 2.34]; AS, 1.54 mL min g [1.41, 1.66]; *P*<0.0001) before AVR. Before AVR, within the AS group, patients with AS-T2D had worse PCr/ATP (AS-noT2D, 1.74 [1.62, 1.86]; AS-T2D, 1.44 [1.32, 1.56]; *P*=0.002) and vasodilator stress MBF (AS-noT2D, 1.67 mL min g [1.5, 1.84]; AS-T2D, 1.25 mL min g [1.22, 1.38]; *P*=0.001) compared with patients with AS-noT2D. Before AVR, patients with AS-T2D also had worse PCr/ATP (AS-T2D, 1.44 [1.30, 1.60]; T2D controls, 1.66 [1.56, 1.75]; *P*=0.04) and vasodilator stress MBF (AS-T2D, 1.25 mL min g [1.10, 1.41]; T2D controls, 1.54 mL min g [1.41, 1.66]; *P*=0.001) compared with T2D controls at baseline. After AVR, PCr/ATP normalized in patients with AS-noT2D, whereas patients with AS-T2D showed no improvements (AS-noT2D, 2.11 [1.79, 2.43]; AS-T2D, 1.30 [1.07, 1.53]; *P*=0.0006). Vasodilator stress MBF improved in both AS groups after AVR, but this remained lower in patients with AS-T2D (AS-noT2D, 1.80 mL min g [1.59, 2.0]; AS-T2D, 1.48 mL min g [1.29, 1.66]; *P*=0.03). There were no longer differences in PCr/ATP (AS-T2D, 1.44 [1.30, 1.60]; T2D controls, 1.51 [1.34, 1.53]; *P*=0.12) or vasodilator stress MBF (AS-T2D, 1.48 mL min g [1.29, 1.66]; T2D controls, 1.60 mL min g [1.34, 1.86]; *P*=0.82) between patients with AS-T2D after AVR and T2D controls at follow-up. Whereas global longitudinal strain, 6-minute walk distance, and NT-proBNP all improved after AVR in patients with AS-noT2D, no improvement in these assessments was observed in patients with AS-T2D.

**CONCLUSIONS:**

Among patients with severe AS, those with T2D demonstrate persistent abnormalities in myocardial PCr/ATP, vasodilator stress MBF, and cardiac contractile function after AVR; AVR effectively normalizes myocardial PCr/ATP, vasodilator stress MBF, and cardiac contractile function in patients without T2D.

**Clinical Perspective**

**What Is New?**

- Patients with severe aortic stenosis (AS) and type 2 diabetes (T2D) show greater impairments in myocardial phosphocreatine to ATP ratio, vasodilator stress myocardial blood flow, and global longitudinal strain compared with patients with severe AS without diabetes and compared with patients with T2D without AS.
- Aortic valve replacement leads to significant improvements in cardiac remodeling, myocardial phosphocreatine to ATP ratio, vasodilator stress myocardial blood flow, global longitudinal strain, and 6-minute walk distance in patients with severe AS alone; in contrast, patients with severe AS and T2D display persistent impairments in these assessments.

**What Are the Clinical Implications?**

- In patients with severe AS and T2D, the efficacy of aortic valve replacement for severe AS is attenuated in reversing preexisting myocardial abnormalities present before the valve replacement, which may be a plausible mechanism for the adverse prognosis after aortic valve replacement in patients with T2D and AS.

Type 2 diabetes (T2D) is present in 22% to 36% of patients with severe aortic stenosis (AS).^1–4^ Compared with patients with AS without T2D (AS-noT2D), patients with AS and T2D (AS-T2D) have higher cardiovascular morbidity and mortality rates after aortic valve replacement (AVR).^4–8^ AS and T2D share common cardiac features of adverse remodeling, abnormal energetics, and reduction in vasodilator stress myocardial blood flow (MBF).^9–12^ These abnormalities may underpin the adverse prognosis observed in patients with AS-T2D.

The relative concentration of phosphocreatine to adenosine triphosphate (PCr/ATP) can be measured noninvasively in the myocardium using ^31^P magnetic resonance spectroscopy (^31^P-MRS).^13^ This provides a sensitive indicator of the myocardial energetic state, reflecting the balance between energy production and use. Cardiovascular magnetic resonance imaging (CMR) is the reference standard for assessment of the myocardial remodeling observed in AS, including left ventricular (LV) volumes, mass, and wall thickness as well as changes in systolic function.^1^ CMR also provides assessment of myocardial fibrosis using late gadolinium enhancement (LGE) and T1 mapping techniques, which provide powerful prognostic information in patients with AS undergoing AVR.^1,14^ CMR perfusion mapping allows pixel-wise quantification of perfusion indices at rest and pharmacologic stress, including MBF in mL min g and myocardial perfusion reserve (MPR).^15^

In this prospective study, ^31^P-MRS, CMR, and 6-minute walk tests were performed before and after AVR in patients with severe symptomatic AS with T2D and patients with severe symptomatic AS who did not have T2D. Two control populations were also recruited. Participants with T2D who did not have AS (T2D controls) underwent identical assessments at baseline and 6 months later, and demographically matched healthy volunteers (HVs) underwent ^31^P-MRS and CMR at a single time point. This study tested the hypothesis that patients with severe AS and T2D (AS-T2D) would have impaired myocardial energetics as reflected by the PCr/ATP ratio and vasodilator stress MBF compared with patients with severe AS but no T2D (AS-noT2D) and that these differences would persist after AVR.

## METHODS

### Study Design and Oversight

This single-center, longitudinal, prospective cohort study complied with the Declaration of Helsinki and was approved by the National Research Ethics Committee (reference 18/YH/0168 for the AS cohorts and reference 18/YH/0168 for HVs with overweight or obesity and longitudinal data of T2D controls). The study was cofunded by the Wellcome Trust (grant 207726/Z/17/Z) and Diabetes UK (18/0005870). The data will be shared on reasonable request to the corresponding author.

### Participants

Adult patients with severe AS who had been referred for surgical AVR (SAVR) or transcatheter AVR (TAVR) were eligible for inclusion. The diagnosis of severe AS was made on the basis of peak aortic forward flow velocity of >4 m/s on valve clinic echocardiography according to current cardiovascular society guidelines.^16,17^ Ninety-five participants with severe symptomatic AS awaiting AVR were prospectively recruited: 65 had severe AS in isolation, and 30 had severe AS-T2D. Data collection was completed between April 2019 and April 2022. Patients with AS-T2D had an established T2D diagnosis according to World Health Organization criteria and were free from known diabetes complications.^18^ Fifteen HVs with normal body weight and 15 overweight HVs were recruited to serve as healthy control cohorts. Fifteen normal-weight and 15 overweight patients with T2D without a history of cardiovascular comorbidities were recruited as a T2D control cohort. All participants for all study cohorts provided written informed consent in line with National Research Ethics Committee requirements (reference 18/YH/0168 for the AS cohorts and reference 18/YH/0168 for HVs with overweight or obesity and controls with T2D).

Controls with and without T2D were contemporary participants and were recruited from primary care general practices in Yorkshire, UK. Electronic health records were screened to assess potential eligibility by primary care physicians. Potentially eligible controls with T2D and healthy controls were then sent study information by postal mail and invited to contact research investigators to discuss study participation.

**Figure 1.**
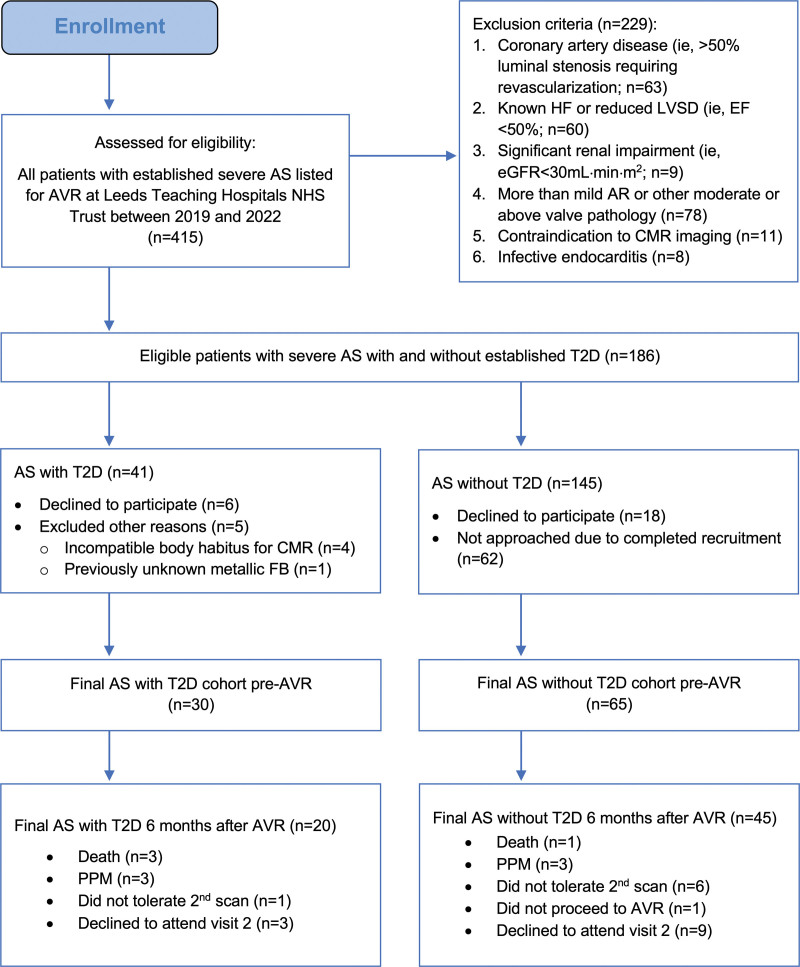
**Consort flow diagram demonstrating the recruitment pathway for study patients with AS.** AR indicates atrial regurgitation; AS, aortic stenosis; AVR, aortic valve replacement; CMR, cardiovascular magnetic resonance imaging; EF, ejection fraction; eGFR, estimated glomerular filtration rate; FB, foreign body; HF, heart failure; LVSD, left ventricular systolic dysfunction; NHS, National Health Service; T2D, type 2 diabetes.

Potentially suitable patients with severe AS were prescreened from AVR waiting lists (SAVR or TAVR) at the Leeds Teaching Hospitals NHS Trust, UK. These SAVR and TAVR lists were reviewed once weekly for new additions. Hospital electronic health records were screened to assess potential eligibility. Potentially eligible patients with AS were invited for a research visit by postal mail (Figure 1). Patients with AS with and without T2D were matched for median surgical risk scores, AS severity, and frailty scores.

Patients with AS underwent CMR, ^31^P-MRS, and 6-minute walk test within 1 month before AVR. Six months after AVR, the surviving participants were invited for repeat assessments. Patients were followed for a median of 13 months after AVR for clinical outcomes by hospital electronic health records. T2D controls underwent identical assessments at baseline and 6 months later; normal-weight and overweight healthy controls were assessed once and did not undergo 6-minute walk testing. The clinical outcomes were monitored only for the patients with AS.

### Exclusion Criteria

Participants were excluded if they had known previous myocardial infarction, previous coronary artery bypass grafting, angioplasty, flow-limiting coronary artery disease (CAD), chronic obstructive lung disease, asthma, tobacco smoking, more than mild bystander valve disease, substantial kidney dysfunction (estimated glomerular filtration rate <30 mL min 1.73 m^2^), permanent atrial fibrillation, known heart failure or reduced LV ejection fraction (EF; <50%), cardiomyopathy (on the basis of infiltrative diseases [eg, amyloidosis], accumulation diseases [eg, hemochromatosis, Fabry disease], or hypertrophic cardiomyopathy), or any contraindication to CMR scanning. Because the research protocol included a 6-minute walk test, potential participants with mechanical or permanent mobility issues were also excluded. All patients listed for AVR had flow-limiting CAD excluded by invasive angiography and previous myocardial infarction excluded by LGE imaging.

Exclusion criteria for T2D controls and healthy controls were known CAD, cardiac surgery, tobacco smoking, amyloidosis, permanent atrial fibrillation, more than mild forms of other heart valve disease, kidney dysfunction (estimated glomerular filtration rate <30 mL·min·1.73 m^2^), or contraindications to CMR.

### Anthropometric Measurements

Height and weight were recorded, and body mass index (BMI) was calculated. Blood pressure was recorded after the participant was seated for >10 minutes. A 12-lead ECG was recorded. A fasting blood sample was taken for assessments of full blood count, estimated glomerular filtration rate, lipid profile, and glycated hemoglobin (HbA1c), insulin, free fatty acid, β-hydroxybutyrate, and NT-proBNP (N-terminal pro-B-type natriuretic peptide) levels.

### Surgical Risk Scores and Frailty Scores

Both Euro Score II and the Society of Thoracic Surgeons risk scores were recorded. As validated measures of frailty and comorbidity, both the Rockwood Frailty Score and the Charlson Comorbidity Index were recorded.

### ^31^P Magnetic Resonance Spectroscopy

^31^P-MRS was performed to assess myocardial PCr/ATP ratio from a voxel placed in the midventricular septum, with patients lying supine and a ^31^P transmitter/receiver cardiac coil (Rapid Biomedical GmbH) placed over the heart on a 3.0 T magnetic resonance imaging system (Prisma; Siemens) as previously described.^19^ ^31^P-MRS is not licensed for scanning patients with a pacemaker; therefore, patients who required a pacemaker implantation after AVR underwent repeat CMR but not repeat ^31^P-MRS.

### Cardiovascular Magnetic Resonance

**Figure 2.**
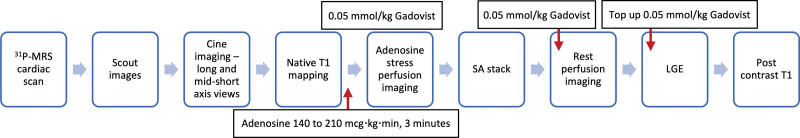
**Multiparametric scan protocol.** Cardiac ^31^P magnetic resonance spectroscopy (MRS) was followed by cardiovascular magnetic resonance imaging, which included cine imaging, native precontrast, and native postcontrast T1 mapping; adenosine stress perfusion imaging; and late gadolinium enhancement (LGE) imaging. SA indicates short axis.

The CMR protocol (Figure 2) consisted of cine imaging using a steady-state free precession sequence, native precontrast and postcontrast T1 mapping, stress and rest perfusion, and LGE imaging.

Native T1 maps were acquired in 3 short-axis slices, using a breath-held modified look-locker inversion recovery acquisition as previously described.^20^ Postcontrast T1 mapping was performed using the same approach 15 minutes after the last contrast injection.

Perfusion imaging used free-breathing, motion-corrected automated in-line perfusion mapping. Adenosine was infused at a rate of 140 µg kg·min for a minimum of 3 minutes according to hemodynamic and symptomatic response, as previously described.^20^ Two trained cardiologists with advanced life support training monitored the patients during adenosine stress imaging. Adenosine stress was tolerated well by all patients during the stress perfusion studies. LGE was performed using a phase-sensitive inversion recovery sequence >8 minutes after contrast administration.

### Quantitative Analysis of ^31^P-MRS and CMR Data

All ^31^P-MRS and CMR postprocessing analyses (except for the perfusion mapping) were performed offline. Image analysis was conducted blind to the study groups (ie, analyzed in batches after the end of each 4-monthly scanning window). Perfusion mapping used artificial intelligence (Gadgetron framework) for instantaneous quantification of perfusion indices rest/stress MBF and MPR.^15^

^31^P-MRS analysis was performed offline by N.J. using software within MATLAB version R2012a (MathWorks) as previously described.^19^

CMR image analysis was performed by N.J., and scan contours were subsequently reviewed by E.L., also blinded to participant details, using cvi42 software (Circle Cardiovascular Imaging). Images for biventricular volumes, function, and LV maximal wall thickness were analyzed as previously described.^21^

Left atrial volume and EF were calculated using the biplane area-length method in the horizontal and vertical long axes as previously described.^21^ Strain measurements were performed using cvi42 Tissue Tracking from the short axis images and the long axis views. Peak diastolic strain rate and global longitudinal strain (GLS) were measured.^21^ T1 maps and extracellular volume (ECV) were analyzed using cvi42 software as previously described.^19^

The LV short axis stack of images was first assessed visually for presence of late gadolinium hyperenhancement, followed by quantification when late gadolinium hyperenhancement was present, as previously described.^22^ Late gadolinium hyperenhancement was defined as areas of signal intensity ≥5 SDs from normal myocardium and was expressed as the percentage of LV mass, quantified in a blinded fashion.

### Six-Minute Walk Test

Participants were instructed to walk along a 30-meter corridor and cover the maximum achievable distance in 6 minutes under the supervision of study investigators with medical training and with experience in conducting the test. At the end of 6 minutes, participants were asked to stop, and the distance walked was measured in meters.

### Clinical Outcomes

Patients were followed for a median of 13 months after AVR for clinical outcomes using electronic health records. The clinical event rates for cardiovascular mortality after AVR and separately the clinical event rates for a composite of all-cause mortality, myocardial infarction, infective endocarditis, and heart failure hospitalization were assessed. For the composite clinical end point, all time-to-event analyses were made based on the first relevant unrefuted event (ie, an event of a particular type was included in the analysis if it had been confirmed in the health care notes).

### Sample Size

A priori sample size calculations were performed based on pilot data (myocardial PCr/ATP ratio and vasodilator stress MBF) obtained from 10 patients with severe AS (5 with and 5 without T2D). These pilot study assessments showed a mean±SD myocardial PCr/ATP ratio of 1.33±0.25 in patients with AS-T2D versus 1.71±0.28 in patients with AS-noT2D. On the basis of these pilot data for the 2 groups, a minimum of 18 patients was needed to be recruited (9 per group) to detect a significant difference in myocardial PCr/ATP ratio between patients with severe AS with and without T2D with 90% power at a 5% significance level on a 2-sample *t* test (calculations performed on ClinCalc.com software). A second sample size calculation was performed for comparisons of vasodilator stress MBF between the 2 groups (AS-T2D, 1.28±0.66 mL min g versus AS-noT2D, 1.89±0.68 mL min g). On the basis of these pilot data for the 2 groups, a minimum of 50 patients was needed to be recruited (25 per group) to detect a significant difference in the vasodilator stress MBF myocardial PCr/ATP ratio between patients with severe AS with and without T2D with 90% power at a 5% significance level on a 2-sample *t* test (calculations performed on ClinCalc.com software). A third sample size calculation showed that 25 patients with AS-noT2D were needed to complete the study and undergo repeat ^31^P-MRS assessments to detect a modest 13% improvement in the myocardial PCr/ATP ratio after AVR based on the pre-AVR pilot data obtained in this group, with 80% power at a 5% significance level on a 2-tailed paired *t* test. These targets were achieved in this study. In addition to these prespecified comparisons in the myocardial PCr/ATP ratio and vasodilator stress MBF, other analyses were performed with due allowance for their exploratory nature. Power calculations were not performed for the exploratory end points.

### Statistical Analysis

Statistical analysis was performed using GraphPad Prism software (version 9.0.0). All data were checked for normality using the Shapiro-Wilk test and presented as mean or median with 95% CIs as appropriate. Categorical data are presented as numbers and percentages and compared with the Pearson χ^2^ test. Comparisons between >2 groups were performed by 1-way ANOVA with post hoc Bonferroni corrections. Differences in nonparametric variables were assessed using a Kruskal-Wallis test. The Student *t* test was used for comparison of normally distributed data sets, and the Mann-Whitney *U* test was used for nonparametric tests for which data were obtained for only 2 groups. Bivariate correlations were performed using the Pearson or Spearman method, as appropriate. Changes between pre-AVR and post-AVR visits were compared using paired Student *t* tests for continuous variables and the Wilcoxon test for nonparametric tests. A 2-sided *P* value of <0.05 was applied as indicating threshold of significance.

A 2-sided log-rank test was used to calculate average event rate ratios and CIs over a median period of 13 months.

## RESULTS

### Participant Demographic and Clinical Characteristics

Between May 2019 and April 2022, 95 patients with severe AS (30 with and 65 without T2D) who were scheduled for TAVR or SAVR were recruited. Demographic, clinical, valvular, and biochemical data for patients with AS are shown in Table 1. Thirty HVs (15 normal weight and 15 overweight) and 30 patients with T2D (15 normal weight and 15 overweight) without AS were controls (Tables 2 and 3). There were no significant differences in age or sex distributions across study groups.

**Table 1.**
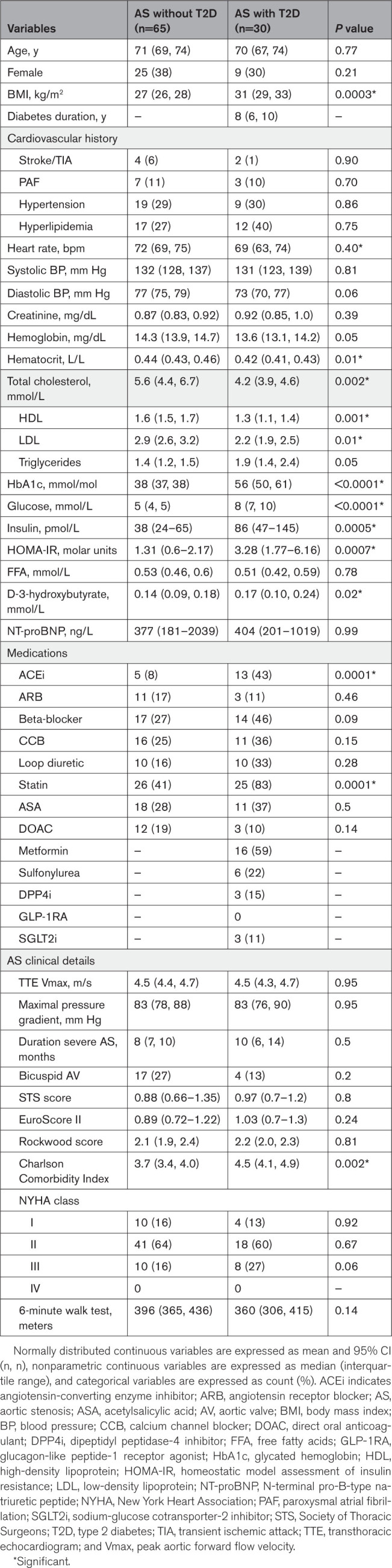
Clinical and Biochemical Characteristics of Patients With AS With and Without T2D

**Table 2.**
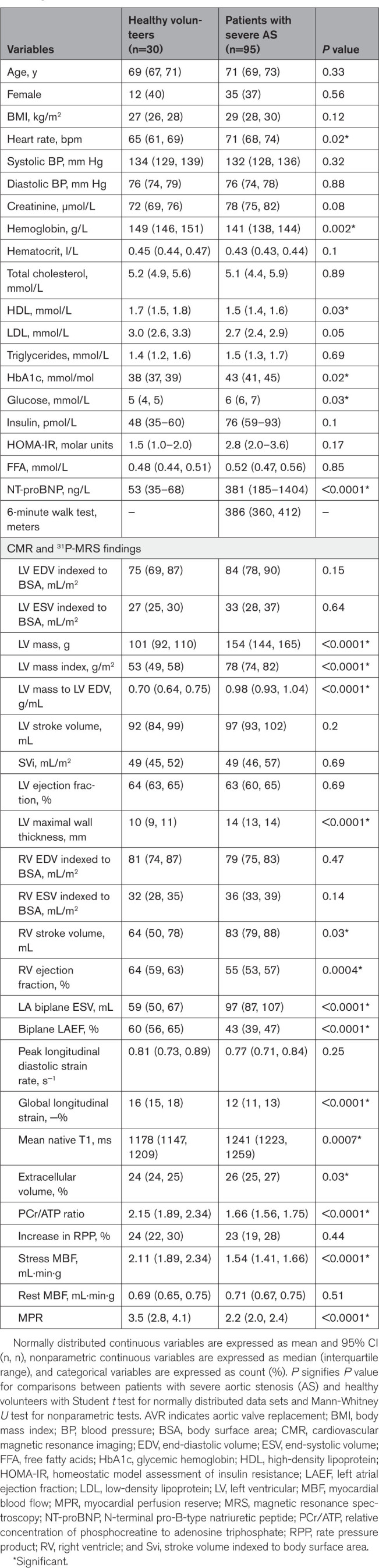
Clinical and Biochemical Characteristics, CMR, and 31P-MRS Comparisons Between Patients With AS and Healthy Volunteers Before AVR

**Table 3.**
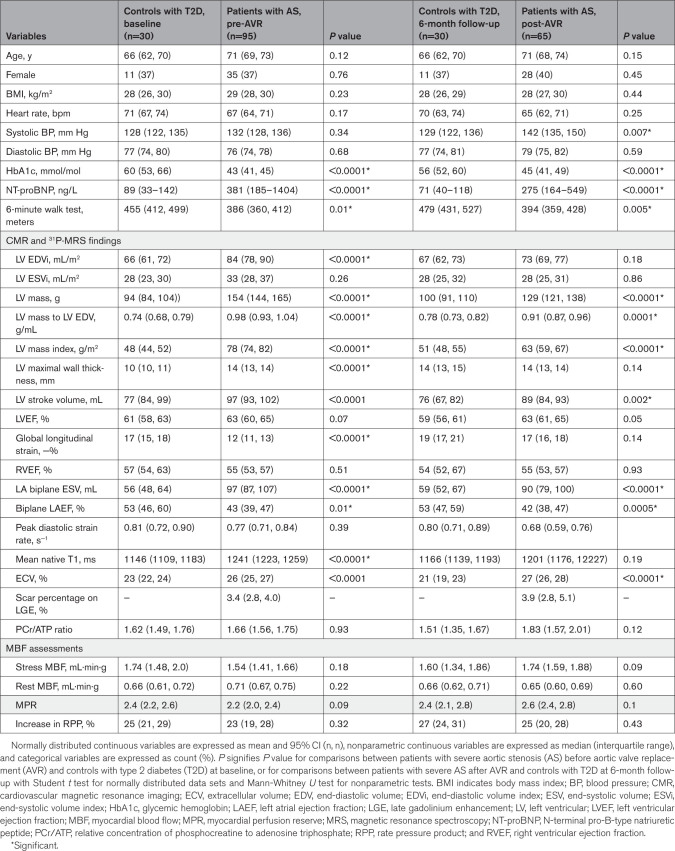
Pre-AVR and Post-AVR Comparisons of Clinical Characteristics, CMR, and ^31^P-MRS Findings Between Patients with AS and Controls with T2D at Baseline and at 6-Month Follow-Up

Compared with HVs, patients with AS had a higher BMI, with elevated fasting glucose, HbA1c, and HOMA-IR (homeostatic model assessment for insulin resistance) levels (Table 2). NT-proBNP levels (median and interquartile range) were also significantly higher in patients with AS (HVs, 42 ng/L [35–66] versus AS, 381 ng/L [185–1404]; *P*<0.0001). Compared with T2D controls, patients with AS showed no significant differences in BMI, but had lower HbA1c levels (Table 3), higher NT-proBNP levels (T2D controls, 89 ng/L [33–142] versus AS, 381 ng/L [185–1404]; *P*<0.0001), and reduced 6-minute walk distance (T2D controls, mean, 455 meters [95% CI, 412, 499] versus AS, mean, 386 meters [95% CI, 360, 412]; *P*=0.01). Compared with T2D controls, patients with AS-T2D showed no significant differences in BMI or HbA1c level (Table 5).

**Table 4.**
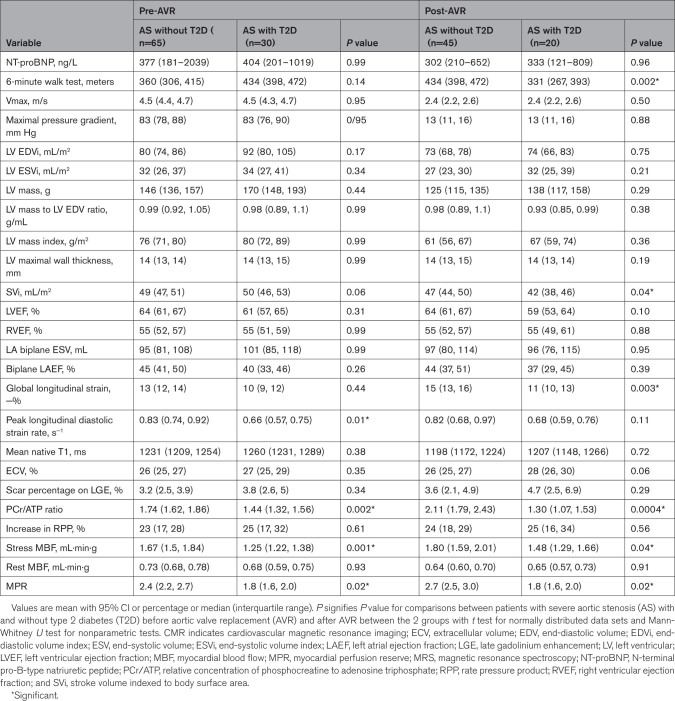
CMR and 31P-MRS Findings in Patients With AS With and Without T2D Before and After AVR

**Table 5.**
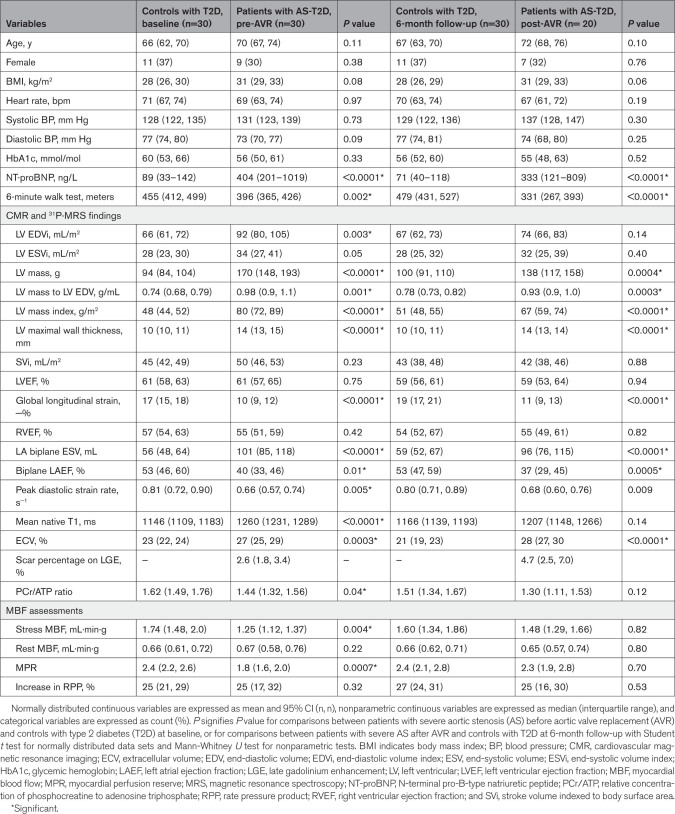
Pre-AVR and Post-AVR Comparisons of Clinical Characteristics and CMR and 31P-MRS Findings Between Patients With AS-T2D and Controls With T2D at Baseline and at 6-Month Follow-Up

Among the patients with AS, those with T2D had higher BMI and fasting glucose and HbA1c levels than patients without T2D (Table 1). Therea were no significant differences in blood pressure or resting heart rate between the 2 groups. A higher proportion of patients with AS-T2D were receiving statin treatment compared with those without T2D, and they consequently had lower total and low-density lipoprotein cholesterol levels. Preoperative 6-minute walk distance and plasma NT-proBNP levels did not differ significantly between the patients with AS who did and did not have T2D.

### Cardiac ^31^P-MRS and CMR Findings

#### Pre-AVR Assessments

Compared with HVs, patients with AS (with and without T2D) had LV concentric hypertrophy with higher LV maximal wall thickness and mass index (Table 2). Whereas they showed impaired GLS values (HVs, mean, −19% [95% CI, 15, 19] versus AS, mean, −12% [95% CI, 11, 13]; *P*<0.0001), there was no significant difference in LV volumes, EF, or peak diastolic strain rate between patients with AS and HVs. Patients with AS also demonstrated impairment in cardiac PCr/ATP (HVs, mean, 2.15 [95% CI, 1.89, 2.34] versus AS, mean, 1.66 [95% CI, 1.56, 1.75]; *P*<0.0001), global stress MBF (HVs, mean, 2.11 mL·min·g [95% CI, 1.89, 2.34] versus AS, mean, 1.54 mL·min·g [95% CI, 1.41, 1.66], *P*<0.0001), and MPR (HVs, mean, 3.8 [95% CI, 2.3, 5.3] versus AS, mean, 2.2 [95% CI, 2.0, 2.4]; *P*<0.0001) compared with HVs. Seventy-eight patients with AS (82%) had noninfarct myocardial scar on LGE imaging, with an average myocardial scar burden comprising 3.4% (95% CI, 2.8, 4.0) of the LV. This was not observed in any of the HVs. Patients with AS also showed a higher myocardial native T1 and ECV fraction as markers of myocardial fibrosis (Table 2).

Compared with T2D controls at baseline, patients with AS had higher LV end-diastolic volumes, maximal wall thickness, and mass index (Table 3). There was no difference in EF or peak diastolic strain rate (Table 3), but patients with AS demonstrated worse GLS values (AS, mean, −12% [95% CI, 11, 13] versus T2D controls, mean, −17% [95% CI, 15, 18]; *P*<0.0001). There were no significant differences in baseline cardiac PCr/ATP (AS, mean, 1.62 [95% CI, 1.49, 1.76] versus T2D controls, mean, 1.66 [95% CI, 1.56, 1.75]; *P*=0.93), stress MBF (AS, mean, 1.74 [95% CI, 1.48, 2.0] versus T2D controls, mean, 1.54 [95% CI, 1.41, 1.66] mL·min·g; *P*=0.18), or MPR (AS, mean, 2.4 [95% CI, 2.2, 2.6] versus T2D controls, mean, 2.2 [95% CI, 2.0, 2.4]; *P*=0.09) between the patients with AS and T2D controls. Patients with AS demonstrated more myocardial scarring (more LGE, higher ECV fraction, and native T1 measurements; Table 3).

**Figure 3.**
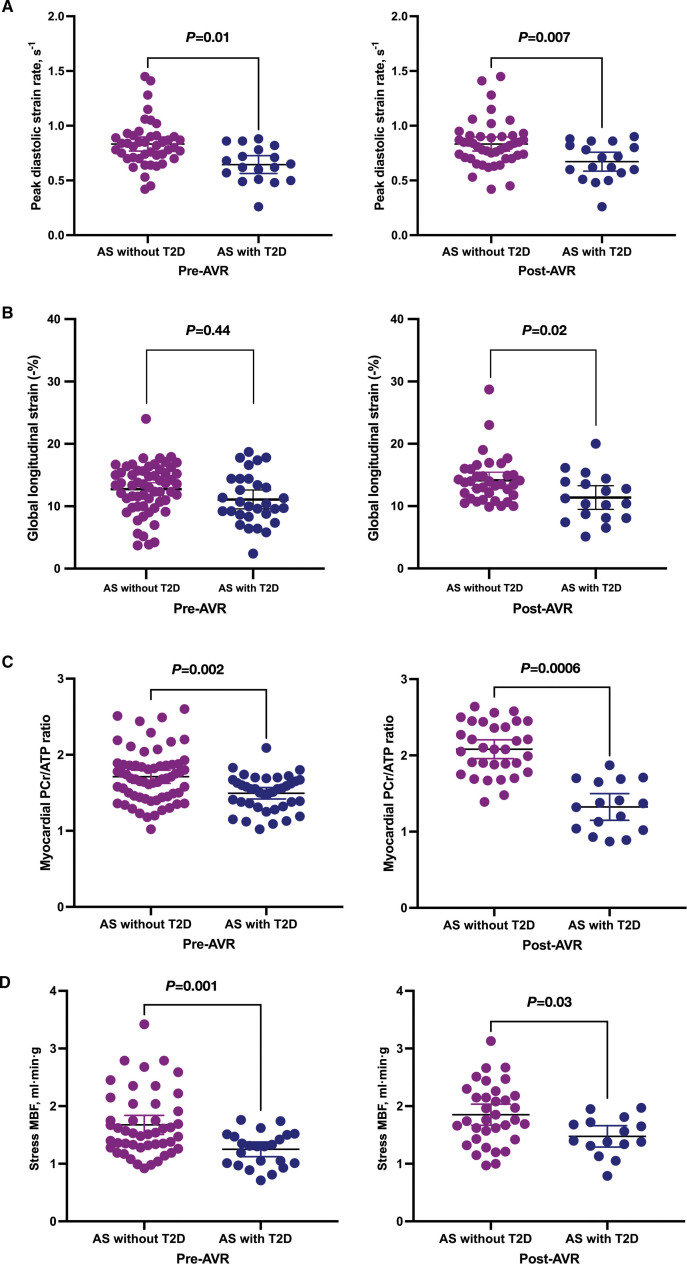
**Differences in peak diastolic strain rate, global longitudinal strain, myocardial PCr/ATP ratio, and stress MBF between patients with AS both with and without T2D before and 6 months after AVR.** Differences in peak diastolic strain rate (s^−1^; **A**), global longitudinal strain (−%; **B**), myocardial phosphocreatine to ATP ratio (PCr/ATP; **C**), and stress myocardial blood flow (MBF; **D**) between patients with aortic stenosis (AS) and type 2 diabetes (T2D) vs patients with AS without T2D before and 6 months after aortic valve replacement (AVR).

Among patients with AS, there was no significant difference in LV volumes, mass index, or maximal wall thickness between those with and without T2D (Table 4). There was no difference in EF (Table 4) or GLS (Figure 3B), but patients with T2D demonstrated an impairment in peak diastolic strain rate (Figure 3A) compared with those without T2D (AS-noT2D, mean, 0.83 s^−1^ [95% CI, 0.74, 0.92] versus AS-T2D, mean, 0.66 s^−1^ [95% CI, 0.57, 0.75]; *P*=0.01). Patients with T2D also had lower cardiac PCr/ATP (AS-noT2D, mean, 1.74 [95% CI, 1.62, 1.86] versus AS-T2D, mean, 1.44 [95% CI, 1.32, 1.56]; *P*=0.002; Figure 3C), vasodilator stress MBF (AS-noT2D, mean, 1.67 [95% CI, 1.5, 1.84] versus AS-T2D, mean, 1.25 [95% CI, 1.22, 1.38] mL·min·g; *P*=0.001; Figure 3D), and MPR values (AS-noT2D, mean, 2.4 [95% CI, 2.2, 2.7] versus AS-T2D, mean, 1.8 [95% CI, 1.6, 2.0]; *P*=0.02). There was no difference in any of the markers of myocardial fibrosis between patients with AS who did and did not have T2D (Table 3).

Compared with T2D controls at baseline, patients with AS-T2D had higher LV end-diastolic volumes, maximal wall thickness, and mass index (Table 5). There was no difference in EF, but patients with AS-T2D demonstrated worse peak diastolic strain rate and GLS values than controls with T2D (Table 5). Patients with AS-T2D had lower cardiac PCr/ATP (AS-T2D, mean, 1.44 [95% CI, 1.30, 1.60] versus T2D controls, mean, 1.66 [95% CI, 1.56, 1.75]; *P*=0.04), vasodilator stress MBF (AS-T2D, mean, 1.25 mL·min·g [95% CI, 1.10, 1.41] versus T2D controls, mean, 1.74 mL·min·g [95% CI, 1.48, 2.0]; *P*=0.004), and MPR (AS-T2D, mean, 1.8 [95% CI, 1.6, 2.0] versus T2D controls, mean, 2.2 [95% CI, 2.0, 2.4]; *P*=0.0007) than T2D controls. T2D controls demonstrated no significant myocardial scarring on LGE and had significantly lower ECV fraction and native T1 measurements than patients with AS-T2D (Table 5).

Subgroup analyses with the control groups stratified by BMI suggested no confounding effect of obesity in the observed differences between patients with AS and the control groups, or between patients with and without T2D (Tables S1 through S3).

#### Six Months After AVR

Similar proportions of patients with AS who did and who did not have T2D underwent TAVR (23% versus 25%, respectively) or SAVR (77% versus 75%, respectively). Six months after AVR, peak aortic valve velocities and mean gradients were similar in the 2 groups (Table 4).

After AVR, patients without T2D demonstrated clear reverse remodeling with reductions in LV mass index compared with pre-AVR measurements (pre-AVR AS-noT2D, mean, 76 g/m^2^ [95% CI, 71, 80] versus post-AVR AS-noT2D, mean, 61 g/m^2^ [95% CI, 56, 67]; *P*=0.0002). By contrast, patients with T2D did not demonstrate significant improvements in LV mass index (Table 4). LVEF remained normal in both groups, with no change observed in either group after AVR. Patients without T2D demonstrated improvements in GLS that were not observed in those with T2D (Figure 3B). The peak diastolic strain rate remained normal in patients without T2D (Figure 3A), whereas it remained impaired with no improvement in patients with T2D.

Cardiac PCr/ATP improved to normal levels after AVR in patients without T2D but did not change in those with T2D. Therefore, cardiac PCr/ATP remained significantly lower in patients with AS-T2D compared with patients with AS but no T2D (AS-noT2D, mean, 2.11 [95% CI, 1.79, 2.43] versus AS-T2D, mean, 1.30 [95% CI, 1.07, 1.53]; *P*=0.0006).

Vasodilator stress MBF improved in both groups after AVR but remained significantly lower in patients with T2D than in those without (AS-noT2D, mean, 1.80 mL·min·g [95% CI, 1.59, 2.0] versus AS-T2D, mean, 1.48 mL·min·g [95% CI, 1.29, 1.66]; *P*=0.03). No change in myocardial fibrosis assessments (LGE%, native T1, ECV fraction) was observed in either group after AVR (Table 4).

After AVR, patients without T2D demonstrated an improvement in 6-minute walking distance (pre-AVR AS-noT2D, mean, 396 meters [95% CI, 365, 436] versus post-AVR AS-noT2D, mean, 434 meters [95% CI, 398, 472]; *P*=0.02) and a reduction in plasma NT-proBNP levels (pre-AVR AS-noT2D, 377 ng/L [181–2039] versus post-AVR AS-noT2D, 302 ng/L [210–652]; *P*=0.04). Again, no improvement in these measures was observed in the patients with T2D (6-minute walking distance pre-AVR AS-T2D, mean, 360 meters [95% CI, 306, 415] versus post-AVR AS-T2D, mean, 331 meters [95% CI, 267, 393]; *P*=0.45; NT-proBNP levels pre-AVR AS-T2D, 404 ng/L [221–1019] versus post-AVR AS-T2D, 333 ng/L [121–809]; *P*=0.17).

Comparisons between post-AVR assessments of all patients with AS versus controls with T2D at the 6-month follow-up showed no significant differences in cardiac PCr/ATP or vasodilator stress MBF (Table 3). Compared with controls with T2D, patients with AS continued to show higher LV maximal wall thickness, mass index, and NT-proBNP levels, even after AVR (Table 3). The 6-minute walk distance also remained significantly shorter, although there was no difference in EF, GLS, or peak diastolic strain rate after AVR (Table 3).

Compared with T2D controls at 6-month follow-up, patients with AS-T2D continued to exhibit higher LV mass index, increased maximal wall thickness, more LGE, and higher ECV fraction 6 months after AVR (Table 5). Whereas there was still no difference in EF, patients with AS-T2D continued to demonstrate worse peak diastolic strain rate (AS-T2D, mean, 0.68 s^−1^ [95% CI, 0.60, 0.76] versus T2D controls, mean, 0.80 s^−1^ [95% CI, 0.71, 0.89]; *P*=0.009) and GLS values (AS-T2D, mean, −11% [95% CI, 9, 13] versus T2D controls, mean, −19% [95% CI, 17, 21]; *P*<0.0001) after AVR compared with T2D controls. After AVR, there were no longer differences in cardiac PCr/ATP (AS-T2D, mean, 1.44 [95% CI, 1.30, 1.60] versus T2D controls, mean, 1.51 [95% CI, 1.34, 1.53]; *P*=0.12), vasodilator stress MBF (AS-T2D, mean, 1.48 mL·min·g [95% CI, 1.29, 1.66] versus T2D controls, mean, 1.60 mL·min·g [95% CI, 1.34, 1.86]; *P*=0.82), or MPR (AS-T2D, mean, 2.3 [95% CI, 1.9, 2.8] versus T2D controls, mean, 2.4 [95% CI, 2.1, 2.8]; *P*=0.70) among patients with AS-T2D versus T2D controls.

### Clinical Outcomes

This preliminary study was not designed to detect clinical outcome differences between patients with AS with and without T2D. However, participants were followed up for a median of 13 months (interquartile range, 10–26 months) after SAVR or TAVR. A total of 7 clinical events (a composite of all-cause death, heart failure hospitalization, infective endocarditis, and myocardial infarction [embolic event 1 week after AVR]) and 4 cardiovascular deaths were observed. There was a higher cumulative incidence of the composite end point in patients with AS who had T2D compared with those who did not (hazard ratio, 7.3 [95% CI, 1.2–45]; *P*=0.03; Table S4). There also appeared to be a higher incidence of cardiovascular death in the AS-T2D group (hazard ratio, 7.6 [95% CI, 0.9–64]; *P*=0.04; Table S4).

## DISCUSSION

Epidemiologic studies have shown that diabetes is associated with LV dysfunction in patients with AS.^23–25^ Moreover, among patients with AS, those with diabetes were shown to have a 2-fold to 3-fold higher risk of death from heart failure, as well as a higher risk of sudden cardiac death.^1,24,26^ As T2D is associated with intrinsic impairments in myocardial energy metabolism and coronary microvascular function,^27–34^ persistent deficits in the myocardial energetic state and abnormalities in MBF even after AVR may be implicated in this process.

In this prospective longitudinal cohort study, severe AS-associated impairments in myocardial PCr/ATP ratio, vasodilator stress MBF, and GLS were amplified in patients with severe AS-T2D, and only these patients showed impairment in diastolic function. Moreover, whereas AVR effectively reversed myocardial impairments in PCr/ATP ratio, stress MBF, and GLS in patients without T2D, those with T2D demonstrated persistent impairments in myocardial PCr/ATP ratio, vasodilator stress MBF, GLS, and diastolic function, as well as continued limitations in exercise capacity after AVR. These findings suggest that post-AVR myocardial recovery is impaired in patients with AS-T2D, and that these residual myocardial abnormalities may explain their adverse prognosis.

The comparisons between patients with AS-T2D pre-AVR and T2D controls at baseline demonstrated that the combined effect of severe AS-T2D is associated with worse impairments in both myocardial PCr/ATP ratio and vasodilator stress MBF before the AVR compared with T2D in isolation. Moreover, the observation that there were no significant differences in myocardial PCr/ATP ratio or vasodilator stress MBF between patients with AS-T2D 6 months after AVR compared with T2D controls at 6-month follow-up indicates that valve intervention can only reverse impairments in myocardial energetics and myocardial vasodilator stress MBF that are related to the AS. Impairments in myocardial PCr/ATP ratio or vasodilator stress MBF related to T2D remain, providing a possible explanation for the adverse prognosis observed in this group, as well as potential treatment targets for future therapeutic intervention.

### Myocardial PCr/ATP Ratio

Myocardial energetic deficit, indicated by a decreased PCr/ATP ratio, is a predictor of mortality,^35^ linked to contractile dysfunction,^35,36^ and a well-recognized complication of T2D^11,12^ and severe AS.^9,37^ Previous studies have demonstrated that there is restoration of myocardial PCr/ATP ratio after AVR,^9,37^ consistent with the findings here in patients without T2D, who demonstrated a 21% relative increase in PCr/ATP ratio compared with pre-AVR measurements. However, this study has shown for the first time that the situation is different for patients with AS-T2D, who display no such improvements in the PCr/ATP ratio after AVR. The study findings also suggest that myocardial PCr/ATP ratio reductions are intrinsic to T2D and detected even in the absence of AS or overweight or obesity, in line with previous studies.^30^ This study has also confirmed that the pressure overload imposed on the left ventricle in AS contributes to an energetic deficit, with patients with AS-noT2D also demonstrating reductions in PCr/ATP ratio (albeit to a lower degree than for patients with AS-T2D), which then improved to similar values to those seen in healthy controls after AVR. Relevant for the interpretation of the impairment in the myocardial PCr/ATP ratio in patients with severe AS, LV hypertrophy in previous studies was shown to be associated with a decrease in the phosphocreatine pool.^38^ In turn, this might reduce the creatine kinase flux and therefore contribute to the reduction in the myocardial PCr/ATP ratio, as recently demonstrated in patients with severe AS.^39^ Supporting this notion, the current study showed improvements in myocardial PCr/ATP ratio after AVR, in parallel with significant regression of LV mass in patients with severe AS-noT2D.

Although for patients with AS-noT2D, the post-AVR normalization of myocardial PCr/ATP was accompanied by significant improvements in GLS, neither PCr/ATP nor GLS improved in patients with AS-T2D. Taken together, these findings may suggest a potential link between energetics and contractile function in both AS groups, which is in line with the knowledge that myocardial contraction is an energy-dependent process, with ATP required in systole for the power stoke of the actin–myosin cross-bridges leading to muscle contraction.

### Myocardial Blood Flow

In line with the findings from this study, previous studies tracking changes in perfusion in patients with AS showed that AVR is associated with restoration of MBF, with improved microcirculatory function resulting from the relief of mechanical obstruction and LV mass regression without accompanying substantial epicardial CAD.^9,37^ However, this study also shows that the combined effects of AS-T2D on MBF may result in lower vasodilator stress MBF and MPR before AVR. In the absence of substantial epicardial CAD, failure to increment MBF during acute increases in cardiac workload may be indicative of coronary microvascular dysfunction, which has been shown previously in both T2D and severe AS.^11,32,40,41^

The findings from this study support the notion that vasodilator stress MBF and MPR reductions are also intrinsic to T2D and detected even in the absence of AS, in line with previous studies.^11,42^ However, these reductions in stress MBF were exacerbated by the combined presence of AS and T2D compared with either disease alone. This finding likely suggests a synergistic insult on the coronary microvasculature between AS and T2D.

### Clinical Implications

Although the findings from this study suggest a mechanistic link between T2D comorbidity and adverse clinical outcomes after AVR, studies are needed to rigorously examine the cause–effect relationship between T2D and adverse outcomes in patients with AS. If a causal link is proven, this may suggest that more intensive lifestyle and diabetes control strategies in patients with AS may protect the myocardium from the synergistic insults observed here with concomitant T2D and AS. Moreover, randomized controlled trials are warranted to investigate whether novel classes of glucose-lowering agents such as glucagon-like peptide-1 receptor agonists and sodium-glucose cotransporter-2 inhibitors can improve myocardial health and clinical outcomes in patients with severe AS-T2D. Myocardial energy metabolism and MBF appear to be promising surrogate end points to test the efficacy of such agents.

### Limitations

This study had several limitations. Diabetes control was suboptimal for all T2D groups (patients with AS-T2D and T2D controls) in the study (average HbA1c 57 mmol/mol) compared with guideline-recommended HbA1c targets (HbA1c <48 mmol/mol),^43,44^ although it was better than the control displayed by the average patient with T2D in the United Kingdom (HbA1c 69 mmol/mol).^45^ Because of the cross-sectional nature of the study, causality of the observed differences cannot be inferred, and the small sample recruited at a single site increases the risk of bias and type I error. Whereas obstructive coronary artery stenosis was excluded using x-ray coronary angiography in all patients with AS, the control groups did not undergo anatomic coronary imaging. Substantial coronary artery disease was deemed to be unlikely in these asymptomatic cohorts, supported by their normal resting ECGs and the absence of myocardial infarction and regional stress perfusion defects on their imaging studies.

Although this study was powered to detect differences in imaging-assessed surrogate markers, it was not designed to detect clinical outcome differences between patients with AS with and without T2D. Indeed, with a relatively small number of events and a short duration of follow-up, the finding that the patients with AS-T2D experienced more clinical events should be interpreted with caution, although it is consistent with the established literature.^1,23–26^ A larger study with a longer follow-up duration will be required to confirm the significance of the observed clinical outcome differences. In addition, the complexity of the imaging protocol, in particular the MRS, may limit its widespread use. Larger multicenter studies are required to confirm our findings to establish the feasibility of the more widespread use of this approach and to investigate subgroups including patients undergoing TAVR versus SAVR.

### Conclusions

Among patients with severe AS, those with T2D demonstrate persistent abnormalities in myocardial PCr/ATP, vasodilator stress MBF, and cardiac contractile function after AVR, whereas AVR effectively normalizes myocardial PCr/ATP, vasodilator stress MBF, and cardiac contractile function in those without T2D.

## ARTICLE INFORMATION

### Sources of Funding

The study was jointly supported by the Wellcome Trust (grant 221690/Z/20/Z) and Diabetes UK (grant 18/0005870). N. Jex receives support from Diabetes UK (grant 18/0005908, Diabetes UK PhD studentship). Dr Levelt acknowledges support from the Wellcome Trust clinical career development fellowship (grant 221690/Z/20/Z), Diabetes UK (grant 18/0005870), and National Institute for Health and Care Research, Leeds Biomedical Research Centre. Dr Valkovič is funded by a Sir Henry Dale fellowship, supported jointly by the Wellcome Trust and the Royal Society (221805/Z/20/Z), and also acknowledges the support of the Slovak Grant Agencies VEGA (2/0003/20) and APVV (19-0032). Funding for open access charge was provided by the Wellcome Trust (grant 221690/Z/20/Z). The views expressed are those of the authors and not necessarily those of the National Health Service, the National Institute for Health and Care Research, or the Department of Health and Social Care.

### Disclosures

None.

### Supplemental Material

Expanded Methods and Results

Tables S1–S4

## Supplementary Material
