# Supplementary material for: Association Between Type 2 Diabetes and Changes in Myocardial Structure, Contractile Function, Energetics, and Blood Flow Before and After Aortic Valve Replacement in Patients With Severe Aortic Stenosis
Source: Circulation. 2023 Sep 25;148(15):1138–53. doi: 10.1161/CIRCULATIONAHA.122.063444 (PMC10558154; doi:10.1161/CIRCULATIONAHA.122.063444)
Supplement: Supplementary file 1 [file cir-148-1138-s001.docx]

**SUPPLEMENTAL MATERIALS**

**Expanded methods**

*Transthoracic echocardiography*

The aortic valve morphology, function, calcification and degenerative changes were assessed by two-dimensional echocardiography using a validated semiquantitative visual scoring system against calcium score derived by multislice computed tomography calcium scoring^46^. This scoring system included criteria on calcification (0 to 4), thickening (0-3), localization of calcific lesion (0-2), leaflet mobility (0-4), with a maximum possible score of 11. The inter-observer variability of the visual assessment scoring of aortic valve morphology, all studies were graded by two independent readers (NJ and HP) blinded to participant details and using the same visual grading approaches.

**Expanded results**

*Transthoracic echo cardiography*

There were no significant differences in semiquantitative visual scores of valve morphology and degenerative changes between the two AS groups (AS with T2DM: 8.38[7.9,8.8] vs AS without T2DM: 8.79[8.3,9.3], p=0.41).

*Transcatheter and surgical aortic valve replacement subgroups*

The patients with aortic stenosis (AS) and type 2 diabetes mellitus (T2DM) referred for transcatheter aortic valve replacement (TAVR) were older (67[63,71] vs 78[74,83] years, p=0.001), had higher Society of Thoracic Surgeons (STS) scores (0.9[0.7,1.1] vs 1,3[0.9,1.8], p=0.01) and Charlson comobidity scores (4.0[3.6,4.3] vs 5.4[4.8,6.0], p=0.0001) than the patients AS with T2DM referred for surgical AVR (SAVR). This was similar for the patients with AS alone undergoing TAVR and SAVR, who were also older (68[66,70] vs 80[77,83], p<0.0001), had a higher STS (0.9[0.8,1.0] vs 2.0[0.9,3.0], p=0.0003) and Charlson scores (3.3[3.0,3.6] vs 4.9[4.2,5.7], p<0.0001) than patients with AS alone referred for SAVR.

However, there was no significant difference in age and STS scores between the two AS groups referred for TAVR or SAVR, with a good match in clinical characteristics including age (AS and T2DM SAVR vs AS alone SAVR, p=0.68; AS and T2DM TAVR vs AS alone TAVR, p=0.40) and STS score (AS and T2DM SAVR vs AS alone SAVR, p=0.60; AS and T2DM TAVR vs AS alone AVR, p=0.34). While the Charlson scores were matched between the two AS groups undergoing TAVR, this comorbidity index was higher in patients with AS alone undergoing SAVR than patients with AS and T2DM (AS and T2DM SAVR vs AS alone SAVR, p=0.008; AS and T2DM TAVR vs AS alone TAVR, p=0.16).

A lower percentage of patients had a history of paroxysmal AF diagnosis pre-AVR (13% for TAVR and 13% SAVR) among the patients with AS and T2DM, than patients with AS alone (38% pre-TAVR, 16% pre-SAVR). After AVR these trends were maintained with a lower relative proportion of T2DM comorbidity patients found to be in AF during follow-up (13% post-TAVR, 21% post-SAVR) than AS patients without T2DM (44% post-TAVR, 24% post-SAVR).

*Interobserver reproducibility of the visual scoring of valvular morphology and degenerative changes*

The agreement between the two readers was good, with correlation of scores r=0.88, p=<0.0001, and with a good level of agreement suggested by the Bland Altman analysis with the bias=-0.146(-1.8,-1.5).

*Impact of obesity on cardiac findings*

Control groups were stratified by BMI into 4 normal-weight healthy controls (n=15), overweight healthy controls (n=15), normal-weight controls with T2DM (n=15), overweight controls with T2DM (n=15), and compared to pre-AVR assessments of patients with AS with (n=30) and without (n=65) T2DM in the Supplementary Tables 4 (clinical characteristics) and 5 (CMR and ^31^P-MRS findings).

Suggesting no confounding impact of obesity these comparisons showed that even against weight-matched healthy volunteers or weight-matched controls with T2DM, patients with AS with and without T2DM exhibit LV concentric hypertrophy with higher LV maximal-wall-thickness, mass, and concentricity index. They also show significant impairment of GLS compared to normal-weight as well as overweight controls with and without T2DM. Across all groups only patients with AS and T2DM showed impairment in peak diastolic strain rate pre-AVR.

Again suggesting no confounding effect of obesity, both AS groups demonstrated significant impairment of PCr/ATP and in vasodilator stress MBF and MPR compared to normal-weight and overweight healthy controls; but not compared to normal-weight or overweight T2DM controls. Regardless of BMI the controls with T2DM also showed significant reductions in myocardial PCr/ATP against the normal-weight and overweight healthy controls. Both AS groups demonstrated significantly lower values in PCR/ATP, vasodilator stress MBF and MPR compared to normal-weight and overweight healthy controls, but not compared to normal-weight and overweight T2DM controls.

While reductions in myocardial PCr/ATP ratio in overweight healthy controls were not detected in this study (supplementary materials Table-3), Rayner and colleagues^47^ in a previous study have demonstrated significant reductions in myocardial PCr/ATP ratio in individuals with obesity, with 12% lower group mean values than the normal-weight healthy controls in that study (which were in the same range as in this study). In this study the normal-weight controls with T2DM showed 23.5% lower PCr/ATP compared to normal-weight healthy controls and 21.6% lower PCr/ATP compared to overweight controls. The discrepancy between the two studies is likely due to slightly differing methodologies. In this study only overweight controls with normal HbA1c and normal fasting glucose were included to ensure undiagnosed T2DM or prediabetes did not confound the study findings. In the study by Rayner et al., HbA1c levels were not assessed but fasting glucose levels in the obesity cohort were significantly elevated compared to normal-weight healthy controls, and HOMA-IR values were at similar range to the normal-weight controls with T2DM in this study, suggesting presence of significant insulin resistance.

**Table S1: Clinical characteristics and biochemistry comparisons between the patients with aortic stenosis and control groups stratified by body mass index**

| **Variable** | **Normal-weight**  **controls**  n =15 | **Overweight**  **Controls**  N=15 | **normal-weight T2DM**  N=15 | **Overweight**  **T2DM**  N=15 | **AS without T2DM**  N=65 | **AS with**  **T2DM**  N=30 | **P value** |
| --- | --- | --- | --- | --- | --- | --- | --- |
| **Age, y** | 71(69,74) | 66(62,69) | 66(59,72) | 66(63,69) | 71(69,74) | 70(67,74) | 0.14 |
| **Female, n (%)** | 5(33) | 7(47) | 6(40) | 6(40) | 25(38) | 9 (30) | 0.18 |
| **BMI, kg/m^2^** | 26(24,27) † | 29(27,30) | 24(23,26) \|\| | 32(29,35) ** | 27(26,28) * | 31(29,33) | **<0.0001** |
| **Heart rate, bpm** | 62(56,67) | 67(61,73) | 66(59,73) | 66(61,70) | 72(69,75) | 69(63,74) | 0.08 |
| **Systolic BP, mmHg** | 136(129,143) | 133(124,141) | 129(119,140) | 127(118,136) | 132(128,137) | 131(123,139) | 0.7 |
| **Diastolic BP, mmHg** | 76(73,79) | 76(72,81) | 77(72,83) | 76(72,80) | 77(75,79) | 73(70,77) | 0.89 |
| **Creatinine, umol/l** | 73(70,77) | 71(64,77) | 74(61,89) | 71(58,84) | 77(73,81) | 81(74,87) | 0.25 |
| **Hemoglobin, g/l** | 149(145,152) | 148(144,153) § | 140(132,149) | 150(143,157) # | 143(139,147) | 136(131,142) | **0.003** |
| **Hematocrit, l/l** | 0.45(0.44,0.47) | 0.46(0.45,0.47) £ | 0.42(0.39,0.44) | 0.46(0.44,0.48) # | 0.44(0.43,0.46) | 0.42(0.41,0.43) | **0.0006** |
| **Total cholesterol, mmol/l** | 5.3(4.8,5.7) † | 5.2(4.4,6) | 5.2(4.4,5.9) | 5.0(4.3,5.7) | 5.6(4.4,6.7) * | 4.2(3.9,4.6) | **0.01** |
| **HDL mmol/l** | 1.7(1.6,1.9) † | 1.5(1.3,1.7) | 1.7(1.3,2.0) | 1.4(1.2,1.5) | 1.6(1.5,1.7* | 1.3(1.1,1.4) | **0.001** |
| **LDL, mmol/l** | 2.9(2.6,3.3) † | 3(2.3,3.7) | 2.9(2.3,3.5) | 2.8(2.2,3.4) | 2.9(2.6,3.2) * | 2.2(1.9,2.5) | 0.05 |
| **TG, mmol/l** | 1.3(1.0,1.5) | 1.5(1.1,1.9) | 1.7(1,2.4) | 3.1(1.4,4.8) | 1.4(1.2,1.5) | 1.9(1.4,2.4) | **0.03** |
| **HbA1c, mmol/mol** | 37(36,39) † | 38(36,40) § | 62(51,73) **Ψ** | 58(50,67) ** | 38(37,38) * | 56(50,61) | **<0.0001** |
| **Fasting glucose, mmol/l** | 5(4,5) † | 5(5,5.5) § | 9(7,10) **Ψ** | 8(7,10) ** | 5(4,5) * | 8(7,10) | **<0.0001** |
| **Insulin, pmol/l** | 29[15-58] † | 67(42,91) | 49(31,67) | 122(33,211) ** | 38[24-65] * | 86[47-145] | **0.0001** |
| **HOMA IR, molar units** | 0.8[0.4-1.4] † | 2.2(1.3,3.0) | 3.2(1.6,4.9) **Ψ** | 7.2(2.0,12.0) ** | 1.3[0.6-2.17] * | 3.3[1.77-6.16] | **<0.0001** |
| **FFA, mmol/l** | 0.48(0.44,0.51) | 0.51(0.3,0.52) | 0.55(0.32,0.78) | 0.51(0.37,0.65) | 0.53(0.46,0.6) | 0.51(0.42,0.59) | 0.98 |
| **D-3-hydroxy-butyrate, mmol/l** | - | - | 0.17(0.06,0.28) | 0.14(0.10,0.18) | 0.14(0.09,0.18) | 0.17(0.10,0.24) | 0.1 |
| **NT- proBNP, ng/l** | 42[35-66] † | 72[40,103] § | 126(73,178) \|\| | 72(45,98) # | 377[181-2039] ‡ | 404[201-1019] | **<0.0001** |
| **6 min walk test, m** | - | - | 456(415,497) | 455(362,548) | 396(365,426) | 361(306,415) | 0.07 |
| * **signifies p<0.05 between the AS without T2DM vs AS with T2DM with Bonferroni correction**  † **signifies p<0.05 between AS with T2DM vs normal weight healthy controls with Bonferroni correction**  ‡ **signifies p≤0.05 between AS without T2DM vs normal weight healthy controls with Bonferroni correction**  § **signifies p<0.05 between AS with T2DM vs overweight controls with Bonferroni correction**  \|\| **signifies p<0.05 between AS with T2DM vs normal weight T2DM controls with Bonferroni correction**  # **signifies p<0.05 between AS with T2DM and overweight T2DM controls with Bonferroni correction**  ** **signifies p<0.05 between AS patients without T2DM vs normal weight T2DM controls with Bonferroni correction**  **Ψ signifies p<0.05 between normal weight T2DM controls and normal weight healthy controls with Bonferroni correction**  **Ω signifies p<0.05 between overweight controls and normal weight healthy controls with Bonferroni correction**  AS indicates aortic stenosis; T2DM, type 2 diabetes mellitus; BMI body mass index; BP, blood pressure; bpm, beats per minute; FFA, free fatty acids; HDL, high density lipoprotein; HbA1c, glycemic hemoglobin; HOMA-IR, homeostatic model assessment of insulin resistance; HV, healthy volunteers; LDL, low density lipoprotein; ng/l, nanograms per liter; m, meter; mmol/l, millimoles per liter; min, minutes; NT-proBNP, n-terminal-pro hormone b-type natriuretic peptide; TG, triglycerides | | | | | | | |

|  |
| --- |

**Table S2: List of medications**

| Variable | Healthy  Volunteers  n =15 | Overweight  Controls  N=15 | normal weight T2DM  N=15 | Overweight  T2DM  N=15 | AS without T2DM  N=65 | AS with  T2DM  N=30 | P value |
| --- | --- | --- | --- | --- | --- | --- | --- |
| ACEi | - | 2(13) | 3(20) | 12(80) | 5(8) | 13(43) | **<0.0001** |
| ARB | - | 2(13) | 3(20) | 3(20) | 11(17) | 3(11) | 0.98 |
| Beta blocker | - | 0(0) | 0(0) | 5(33) | 17(27) | 14(46) | **0.0005** |
| CCB | - | 0(0) | 3(20) | 3(20) | 16(25) | 11(36) | 0.06 |
| Loop diuretic | - | 0(0) | 0(0) | 0(0) | 10(16) | 10(33) | **0.001** |
| Statin | - | 5(33) | 10(67) | 15(100) | 26(41) | 25(83) | **<0.0001** |
| ASA | - | 1(7) | 6(40) | 6(40) | 18(28) | 11(37) | 0.15 |
| DOAC | - | 0(0) | 0(0) | 0(0) | 12(19) | 3(10) | 0.08 |
| Metformin | - | - | 10(67) | 12(80) | - | 16(59) | 0.4 |
| Sulfonylurea | - | - | 3(20) | 6(40) | - | 6(22) | 0.38 |
| DPP4i | - | - | 4(27) | 7(47) | - | 3(15) | **0.04** |
| GLP-1RA | - | - | 0(0) | 0(0) | - | 0(0) | - |
| SGLT2i | - | - | 1(7) | 1(7) | - | 3(11) | 0.84 |
| ACEI, angiotensin converting enzyme inhibitor; ARB, angiotensin receptor blocker; CCB, calcium channel blocker; ASA, aspirin; DOAC, direct oral anticoagulant; DPP4i, dipeptidyl peptidase – 4 inhibitor; GLP-1RA, glucagon-like peptide-1 receptor agonist; SGLT2i, sodium glucose co-transporter 2 inhibitor. | | | | | | | |

**Table S3: Baseline CMR and ^31^P-MRS parameters comparisons with controls stratified by body mass index**

| **Variable** | **Normal-weight**  **controls**  n =15 | **Overweight**  **Controls**  N=15 | **normal weight T2DM**  N=15 | **Overweight**  **T2DM**  N=15 | **AS without T2DM**  N=65 | **AS with**  **T2DM**  N=30 | **P value** |
| --- | --- | --- | --- | --- | --- | --- | --- |
| **LV end-diastolic volume indexed to BSA, ml/m^2^** | 78(69,87) | 72(66,79) | 70(61,79) | 62(58,67) # | 80(74,86) **^** | 92(80,105) | **0.0005** |
| **LV mass index, g/m^2^** | 55(47,62) † | 52(46,58) § | 48(41,55) \|\|** | 48(44,53) # **^** | 76(71,80) ‡ | 80(72,89) | **<0.0001** |
| **LV mass to LV end-diastolic volume, g/ml** | 0.66(0.58,0.73) † | 0.72(0.64,0.81) § | 0.69(0.62,0.76) \|\|** | 0.79(0.69,0.89) #**^** | 0.99(0.92,1.05) ‡ | 0.98(0.89,1.1) | **<0.0001** |
| **LV ejection fraction, %** | 65(63,66) | 64(61,67) | 61(57,65) | 63(61,66) | 60(56,64) | 61(58,64) | 0.29 |
| **LV maximal wall thickness, mm** | 10(9,11) † | 10(9,11) § | 10(9,11) \|\|** | 11(9,12) #**^** | 14(13,14) ‡ | 14(13,15) | **<0.0001** |
| **LA biplane end-systolic volumes, ml** | 72(60,85) | 61(49,72) § | 60(46,74) \|\| | 52(43,61) #**^** | 95(81,108) ‡ | 101(85,118) | **<0.0001** |
| **Biplane LA EF, %** | 59(51,66) † | 61(55,68) § | 52(41,64) | 53(43,62) | 45(41,50) | 40(33,46) | **0.0002** |
| **Peak diastolic strain rate, s^-1^** | 0.79(0.68,0.9) | 0.83(0.71,0.95) | 0.81(0.71,0.90) | 0.81(0.64,0.98) | 0.83(0.74,0.92) | 0.66(0.57,0.75) * | **0.04** |
| **Global longitudinal strain, (-%)** | 17(15,19) † | 15(13,17) § | 18(15,20) \|\|** | 16(14,17) # | 13(12,14) ‡ | 10(9,12) | **<0.0001** |
| **Native precontrast T1, (ms)** | 1209(1163,1255) | 1166(1118,1214) § | 1146(1093,1199) \|\|** | 1147(1087,1208) #**^** | 1231(1209,1254) | 1260(1231,1289) | **<0.0001** |
| **Extracellular volume, (%)** | 25(24,26) | 24(22,25) | 24(22,26) | 22(20,24) #**^** | 26(25,27) | 27(25,29) | **0.0009** |
| **Indexed extracellular volume, ml/m^2^** | 11[8,15] †**^,^**‡ | 10[9,12] § | 10[8,13] \|\|** | 9[9,10] #**^** | 19[17,22] | 22[18,25] | **<0.0001** |
| **LGE, (%)** | - | - | - | - | 2(2.5,3.9) | 3.8(2.6,5) | 0.34 |
| **Increase in RPP, %** | 25(17,30) | 24(18,29) | 25(18,30) | 26(19,32) | 23(17,28) | 25(17,32) | 0.54 |
| **Stress MBF, ml/min/g** | 2.14(1.69,2.58) † | 2.08(1.8,2.4) § | 1.73(1.4,2.1) | 1.75(1.3,2.2) | 1.67(1.5,1.84) | 1.25(1.22,1.38) * | **<0.0001** |
| **Rest MBF, ml/min/g** | 0.67(0.58,0.73) | 0.72(0.62,0.82) | 0.69(0.63,0.76) | 0.63(0.53,0.72) | 0.73(0.68,0.78) | 0.68(0.59,0.75) | 0.28 |
| **MPR** | 3.8(2.3,5.3† | 3.2(2.7,3.6) § | 2.3(2,2.6) | 2.5(2.2,2.7) | 2.4(2.2,2.7) ‡ | 1.8(1.6,2.0) * | **<0.0001** |
| **PCr/ATP ratio** | 2.17(1.9,2.5† | 2.12(1.9,2.4) § | 1.66(1.5,1.8) Ψ | 1.56(1.3,1.8) ∇ | 1.74(1.62,1.86) ‡ | 1.44(1.32,1.56) * | **<0.0001** |
| * **signifies p<0.05 between the AS without T2DM vs AS with T2DM with Bonferroni correction**  † **signifies p<0.05 between AS with T2DM vs normal weight healthy controls with Bonferroni correction**  ‡ **signifies p≤0.05 between AS without T2DM vs normal weight healthy controls with Bonferroni correction**  § **signifies p<0.05 between AS with T2DM vs overweight controls with Bonferroni correction**  \|\| **signifies p<0.05 between AS with T2DM vs normal weight T2DM controls with Bonferroni correction**  # **signifies p<0.05 between AS with T2DM and overweight T2DM controls with Bonferroni correction**  ** **signifies p<0.05 between AS patients without T2DM vs normal weight T2DM controls with Bonferroni correction**  **^ signifies p<0.05 between AS patients without T2DM and overweight T2DM controls with Bonferroni correction**  **∇ signifies p<0.05 between overweight T2DM controls and normal weight healthy controls with Bonferroni correction**  **Ψ signifies p<0.05 between normal weight T2DM controls and normal weight healthy controls with Bonferroni correction**  **Ω signifies p<0.05 between overweight controls and normal weight healthy controls with Bonferroni correction**  Values are means or medians with 95% confidence intervals. AS indicates aortic stenosis; T2DM, type 2 diabetes mellitus; HV, healthy volunteer; BSA, body surface area; LV, left ventricle; RV, right ventricle; LV, left ventricular; LA, left atrial; LA EF, left atrial ejection fraction; LGE, late gadolinium enhancement; PCR, phosphocreatine; ATP, adenosine tri-phosphate; RPP, rate pressure product; MBF, myocardial blood flow; MPR, myocardial perfusion reserve; GLS, global longitudinal strain; ms, miliseconds; SVi, stroke volume indexed to body surface area. | | | | | | | |

**Table S4: Clinical events following aortic valve replacement**

| **Clinical Outcomes** | **Aortic stenosis without T2DM**  (n=65) | **Aortic stenosis with T2DM**  (n=30) | |
| --- | --- | --- | --- |
| **Cardiovascular death, n (%)**  **HF Hospitalization, n (%)** | 1 (2)  0 (0) | 3 (11)  1 (4) | |
| **Infective endocarditis, n (%)** | 0 (0) | 1 (4) | |
| **Myocardial infarction, n (%)** | 0 (0) | 1 (4) | |
| T2DM indicates type 2 diabetes mellitus; AS, aortic stenosis; n, number; HF, heart failure; AF, atrial fibrillation. | | | |
|  | | |  |
